# Association of TERT promoter mutation with oral squamous cell carcinoma: a systematic review and meta-analysis

**DOI:** 10.1038/s41598-025-98133-6

**Published:** 2025-04-30

**Authors:** Monal Yuwanati, Sachin Sarode, Gargi Sarode, Amol Gadbail, Shailesh Gondivkar, Akhilanand Chaurasia, Dhara Dwivedi, Sara Delgadillo-Barrera

**Affiliations:** 1 Saveetha Institute of Medical and Technical Sciences, Saveetha Dental College and Hospitals, Saveetha University, Chennai, 600077 India; 2 Dr. D.Y. Patil Dental College & Hospital, Dr. D.Y. Patil Vidyapeeth, Maharashtra State, Pune, India; 3 Department of Dentistry, Shree Bhausaheb Hire Government Medical College & Hospital, Dhule, Maharashtra India; 4 Government Dental College & Hospital, Nagpur, Maharashtra State India; 5 Department of Oral Medicine and Radiology, King George’s Medical University, Lucknow, India; 6 Department of Dental Medicine, College of Medicine and Health Sciences, Hawassa University, Hawassa, Ethiopia; 7 Grupo de Investigación Básica y Aplicada en Odontologia - IBAPO, Facultad de Odontologia, Universidad Nacional de Colombia, Bogotá, Colombia

**Keywords:** OSCC, Mutation, Telomerase reverse transcriptase, C228T, C250T, C228A, Telomere, Promoter, Dental, Cancer, Molecular biology, Health care, Medical research, Oncology

## Abstract

The telomerase reverse transcriptase (TERT) promoter mutations have been linked to the prognosis and survival of several cancers; however, it is still debatable in case of oral squamous cell carcinoma. The exact reason for it is currently unknown. However, considering its role in cell survival and proliferation it is imperative to evaluate the association between TERT promoter mutations and OSCC. The systematic review and meta-analysis were carried out to gather evidence for TERT promotor mutation in OSCC. Electronic databases along with grey literature were searched for relevant studies. We used fixed- or random-effect models to calculate pooled proportion, estimated odds ratios or standardized mean differences, and corresponding 95% confidence intervals (CIs). We included 13 eligible studies incorporating 816 cases. The average frequency of TERT promotor mutation was 46.1% (0.46, 95% CI, 0.33, 0.60). However, TERT promoter mutations were not associated with gender, habit (smoking and betel nut), nodal involvement, metastases, and TNM stage. C228T (189/287), C250T (73/287), C228A, and other variants of TERT mutation were frequently detected TERT mutation variant in OSCC. TERT promoter mutations could be considered as biomarkers assisting in risk stratification and prognostic prediction.

**Supplementary Information:**

The online version contains supplementary material available at 10.1038/s41598-025-98133-6.

## Introduction

Oral squamous cell carcinoma (OSCC) is responsible for millions of deaths in the world^1^. Even with chemotherapy and radiotherapy, it has a compromised prognosis after surgery^2^. Further, the 5-year survival rate has marginally improved over recent years^1^. This is mostly because of excessive self-replication of tumor cells, causing an increase in tumor size, and eventually metastasis. The rate of tumor development, or the time it takes for the tumor to increase in size has been linked to a poorer prognosis^3–6^. Each cell has a limited propensity to divide depending upon telomere length. Telomeres shorten with each cell division, resulting in senesce or apoptosis. Telomere length is determined by the Telomerase Reverse Transcriptase (TERT) enzyme’s activity. TERT is responsible for the formation of telomere DNA repeats at the end of each chromosome. TERT activity is often absent in normal cells, except for those that require continuous division. The absence of TERT activity in normal cells results in limited growth potential and telomere shortening. However, increase in TERT activity may lead to unrestricted self-renewal of cells, one of the hallmarks of cancer. TERT activity can be altered through various mechanisms such as DNA methylation or mutation in mRNA involved in either the formation or degradation of TERT^7–9^.

Several tumors, including OSCC have shown an increase in TERT activity through promotor mutation as well as expression in tumour cells^10,11^. Further, it has been detected in precancerous lesions, suggesting an early indication of malignant transformation^3,12–14^. However, studies have conflicting reports regarding the association between TERT mutation and OSCC. Boscolo-Rizzo et al.^15^ found no significant association between TERT promoter status and overall survival. In contrast, Chang et al.^16^ found a strong association of TERT promotor mutation with progression. TERT’s association with cell proliferation could play an important role in tumour growth and progression. Hence, it is important to analyse its association with OSCC.

## Methods

The systematic review and meta-analysis were reported according to Preferred Reporting Items for Systematic Reviews and Meta-Analyses checklist (PRISMA)^17^. The Systematic review and meta-analysis were registered *priori* in PROSPERO database (CRD42021246459).

### Searches strategy

The PubMed, Scopus, Web of Science, Google Scholar, EMBASE, and Cochrane Library databases were searched to retrieve the relevant articles. Search strategies included a combination of keywords, MeSH (Medical Subject Headings), and glossary terms relevant to any published studies of TERT in OSCC. The search was carried out with no restrictions on year of publication or publication language. The following search strategy was used: (((Squamous cell carcinoma) OR “OSCC”) OR “Head and Neck cancer”) AND (((C250T) OR C228T) OR ((telomerase reverse transcriptase) OR TERT)). The Google Scholar results were sorted by relevance, and the first 100 results were screened. Search was repeated in all above-mentioned databases to find an additional published paper since the last search (last search 19 August 2023). The reference list of all the selected articles was screened manually to identify additional studies left out in the initial search.

## Inclusion and exclusion criteria

The studies were selected on the following inclusion criteria based on PICO framework (Supplementary Table S1): (1) Studiers evaluating TERT promotor mutation in subjects with and /or without OSCC; (2) Assessing the clinicopathological and prognostic roles of TERT promoter mutation in OSCC patients; (3) Providing sufficient data to calculate the measures of association (Hazard ratio or relative risk (RR) or odd ratio (OR) with 95% confidence intervals (95% CI)). Reviews, correspondence, letters, or case reports were excluded. Additionally, studies with overlapping data from the same sample of patients from the same hospitals were excluded.

## Data extraction (selection and coding)

Two authors screened the full text of the included article and extracted the following data: first author, year of publication, study design, country, mean age, gender, number of TERT promotor mutation carriers and non-carriers, TERT mutation detection method, clinicopathological factors, and duration of follow up. Any disagreement was resolved by discussion until consensus was reached or by consulting a third author.

## Assessment of risk of bias

Risk of bias and quality assessment were evaluated according to the Risk of Bias in Non-randomized Studies - of Interventions (ROBINS-I)^18,19^. Two authors performed the quality and risk of bias assessments. The studies were grade as low, moderate, serious or critical risk of bias.

## Data synthesis and meta-analysis

All statistical analyses were performed using Review Manager (Review Manager (RevMan)) [computer program]: Version 5.4. Copenhagen: The Nordic Cochrane Centre, The Cochrane Collaboration, 2014). The included studies were analysed quantitatively and qualitatively. The heterogeneity of studies was assessed by the chi-square test (*P* < 0.1 indicating statistical significance) and I² statistic (a quantitative measure of inconsistency among studies). It was decided to use the random-effect model to pool data only if heterogeneity exceed > 50%. In case of dichotomous outcomes, OR with 95% confidence intervals was calculated by using a fixed effect model (Mantel-Haenszel method) or a random effect model (DerSimonian and Laird method). Subgroup analysis for gender (male vs. female), recurrence (positive vs. negative), disease-free survival, tumour stage (III/IV vs. I/II), and OSCC WHO grade (III/IV vs. I/II) was planned if required data are available. Pooled HR and 95% CI for 5-year survival and association of TERT promotor mutation were calculated for 5-year survival and association of TERT promoter mutation. If survival data or HR were not available, data were extracted with the Kaplan-Meier curves with the help of Engauge Digitizer version 4.1 (http://digitizer.sourceforge.net/). The effect estimated the occurrence of OSCC in individuals with exposure (TERT mutation) versus individuals without exposure (absence of TERT mutation) (OR). Comparison data were available in only two studies.

## Results

### Literature searches and characteristics of included studies

**Fig. 1:**
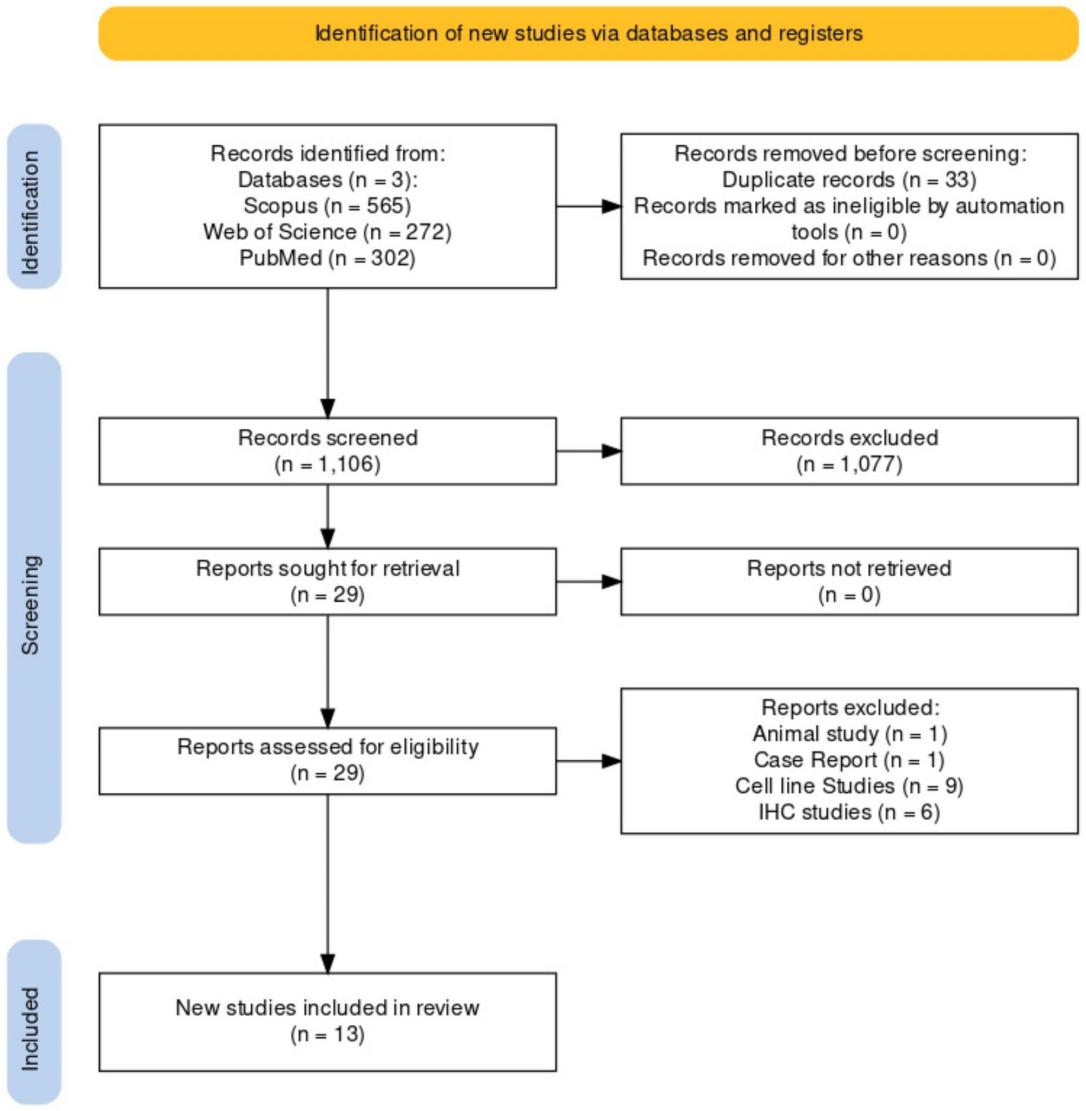
Search, screening, selection, and finalization process used in the TERT in OSCC systematic review and meta-analysis (PRISMA flowchart).

The searcher process is depicted in PRISMA flow chart (Fig. 1). The search yields 746 results from three databases, with 33 duplicates. Initial screening resulted in the removal of 654 non-relevant articles. Finally, 46 were excluded after reading the full text article. Only 13 articles were found eligible for qualitative and quantitative analysis^15,16,21–31^. Detail characteristics were presented in Table 1. Most commonly studied population was the USA^21,27,29,31^, followed by Italy^15,26,30^. One study in Indian, Brazilian, Taiwanese, Turkish population^16,24,28^. Three studies^15, 29^ used control samples. Control samples were either adjacent mucosa or normal mucosa, one study^16^ did not provide details for an unknown control sample. Polymerase chain reaction (PCR), sanger sequencing, pyrosequencing and Next generation sequencing (NSG) are used for the identification of TERT promotor mutations.

**Table 1:** Summary of study characteristics of 13 studies included in systematic review and meta-analysis of TERT promotor mutation in oral squamous cell carcinoma.

Sr no	Author (Year	Country	Study Design	Gender (Female)	Mean Age (years)	Habits (*n*)	Nodal Involvement (N0)	Metastasis (M0)	HPV+ (-)	TNM stage (T1/T2)	Histopathology Grade (G1/G2)	TERT promotor mutation variant (C228T/ C250T)	Mutation Prevalence (% (TERT +/Total))
1.	Killela et al. (2013)	USA	Cross-sectional	22/18	53.85	Tobacco (24)	-	-	2 (6)	-	-	10/2	17.1% (12/40)
2.	Cheng et al. (2015)	USA	Cross-sectional	-	-	-	-	-	-	-	-	2/0	16% (2/12)
3.	Vinoth Kumar et al. (2015)	India	Cross-sectional	35/6	50.27 ± 11.84	Tobacco (30) Betel (13) Alcohol (17)	9 (2)	2 (39)	0 (41)	35 (2)	7 (34)	9/4	31.7% (13/41)
4.	Chang et al. (2017)	Taiwan	Case-control	182/19	52.8 ± 11.3	Tobacco (167) Betel (156) Alcohol (150)	72 (129)			119 (82)	26 (175)	104/26	64.67% (130/201)
5.	Morris et al. (2017)	USA	Cross-sectional	-	-	Smoker (10)	-	6/9	2/12	-	-	4/3 C228A (5)	85% (12/14)
6.	Annunziata (2018)	Italy	Cross-sectional	-	-	-	-	-	-	-	-	3/2	60% (9/15)
7.	Schwaederle M et al. (2018)	USA	Cross-sectional	-	-	-	-	-	-	-	-	-	28.57% (8/28)
8.	Arantes et al. (2020)	Brazil	Cross-sectional	-	-	-	-	-	-	-	-	5/17	31.88% (22/69)
9.	Paolo Boscolo‑Rizzo et al. (2020)	Italy	Case-control	-	66 (range 28 − 27) Median	-	-	-	-	-	-	8/2	37% (10/27)
10.	Yilmaz et al. (2020)	Turkey	Cross-sectional	41/36	59.89 ± 15.50	Smoker (46) Alcohol (11)	-	-	-	-	-	51/21 C228A (5)	75.49% (77/102)
11.	Yu et al. (2021)	USA	Cross-sectional	27/68	57 (28–89)	-	-	-	-	-	-	-	81.08% (60/74)
12.	Moreira et al. (2021)	France	Cross-sectional	59/92	64 (23–91). Median	Smoker (73) Alcohol (48)	-	-	-	-	-		50.33% (76/151)
13.	Giunco et al. (2021)	Italy	Cross-sectional	81/63	65 (54–74) Median	Smoker (89) Drinker (60)	47 (97)	-	-	99 (T1/T2); T3/T4 (45)	104 (G1-G2); 35 (G3)	29/16	31.25% (45/144)

## Association of presence of TERT promotor mutation and OSCC

**Fig. 2:**
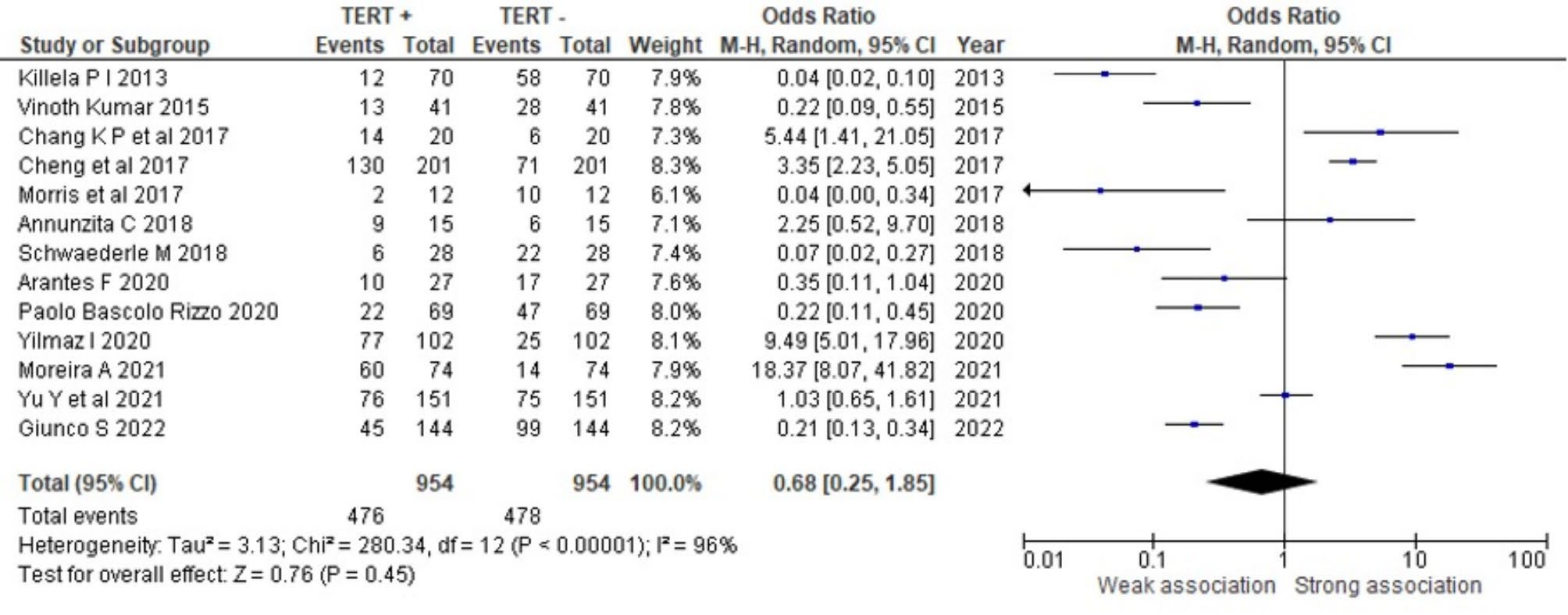
Pooled effect estimates of association of presence of TERT promotor mutation and OSCC.

Figure 2 shows the OR of presence of TERT promotor mutation in OSCC in the 13 studies included. The overall estimate showed that OSCC had an OR of 0.68 (0.25-1.85; I^2^: 96%, *P* = 0.45) for the probability of having TERT promotor mutation.

### TERT promotor mutation in OSCC and normal mucosa

**Fig. 3:**
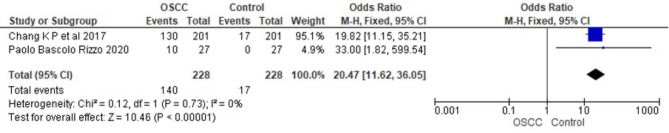
Pooled effect estimates of TERT mutation in OSCC and Normal tissue.

The meta-analysis showed that OSCC had OR 20.47 (11.62–36.05; I^2^: 0%, *P* = 0.00001] of probability of presence of TERT promotor mutation than normal mucosa (Fig. 3). However, this should be interpreted carefully since it is derived from only two studies. Well-designed case-control studies are required to confirm this finding.

### Prevalence/frequency of TERT promotor mutation in OSCC

**Fig. 4:**
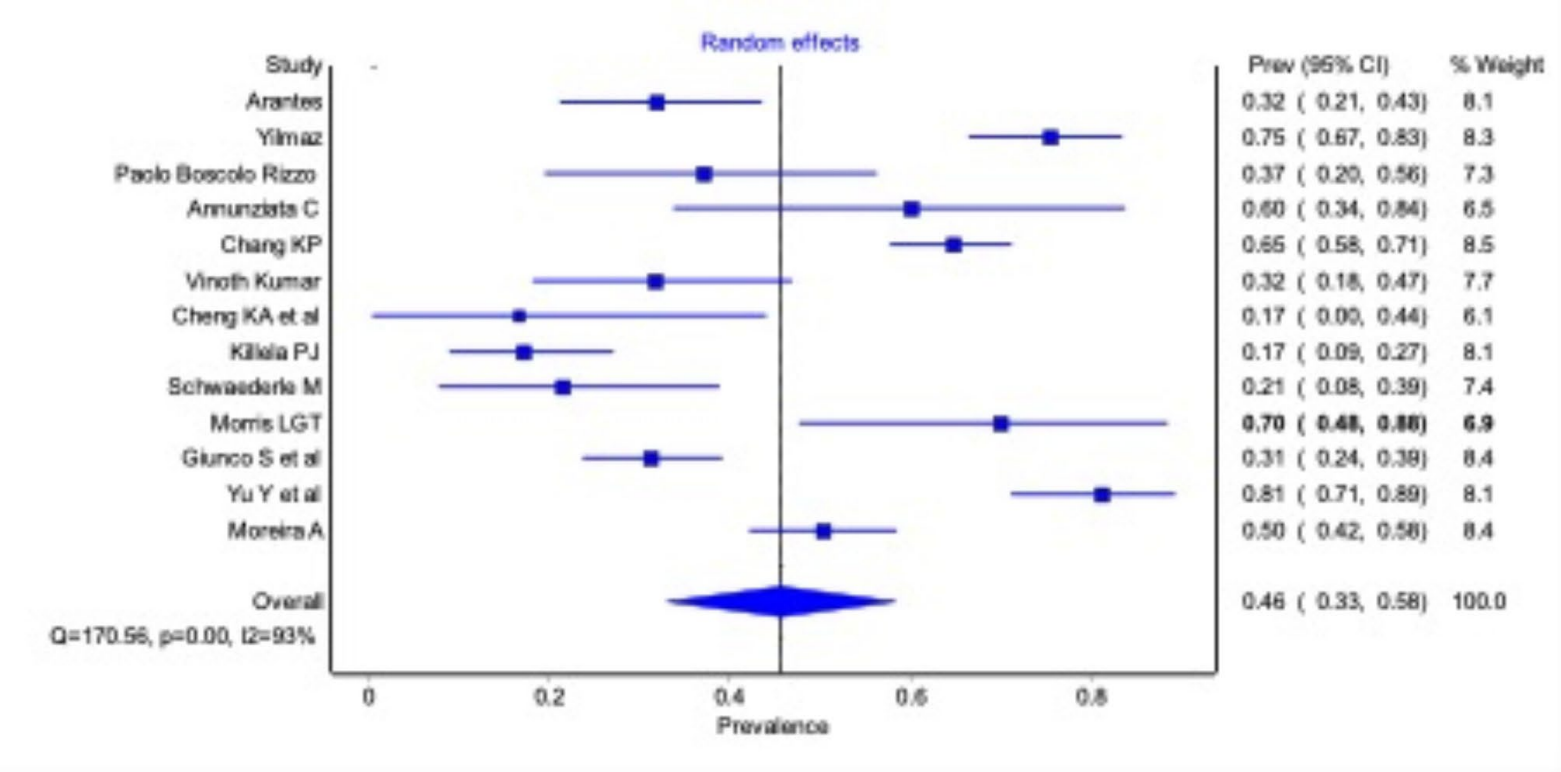
Prevalence/frequency of TERT promotor mutation in OSCC.

Twelve studies estimated the prevalence of TERT promotor mutations in patients. The pooled prevalence for TERT promotor mutation was 46.1% (0.46, 95% CI, 0.33, 0.60) (Fig. 4). In the American population, the pooled prevalence was 32.08% (95% CI, 14.73, 49.43) ranging from 19.04% (95% CI, 13.43, 24.65) to 85.00% (95% CI, 40.74, 129.26). Pooled TERT prevalence for Rest of world population was 46.62% (95% CI, 27.52, 65.72) ranging from 16.00 (95% CI, 7.24, 24.76) to 75.49% (95% CI, 60.95, 90.03). C228T, C250T, C228A, and other variants of TERT mutation were also reported in these studies. Out of these, C228T (189/287) was the most frequently detected TERT mutation variant, followed by C250T (73/287).

### TERT promoter mutations and clinicopathological features of OSCC

A TERT promotor mutation was analysed in 695 OSCC patients (Table 1). Most studies included the oropharynx, tonsil, and laryngeal SCC in cohort to evaluate the TERT promotor mutation. Yilmaz et al.^24^, Giunco et al.^30^, Vinothkumar et al.^25^, Chang et al.^16^, Schwaederle et al.,^27^ Arantes et al.^28^, and Paolo Boscolo‑Rizzo et al.^15^ compared the TERT promotor mutation and clinicopathological parameters. However, Yilmaz et al.^24^, Vinoth Kumar et al.^25^, Giunco et al.^30^, Chang et al.^16^, Arantes et al.^28^ only provided clinicopathological features specifically for OSCC. In that also, only Giunco et al.^30^, Chang et al.^16^, Yilmaz et al.^24^ provided TERT promoter mutation status for clinicopathological parameter separately for OSCC (Supplementary Table S2). In oral cavity SCC, tongue (83/172), floor of mouth (19/53), and buccal mucosa (19/31), and gingiva (7/26) were frequently associated with TERT promotor mutation. Among clinicopathological features, except betel nut chewing habit, gender (female vs. male), smoking, alcohol, age (< 50 year vs. > 50 years), T stage (T1/T2 vs. T3/T4), Node involvement (N0 vs. N1), extracapsular spread (- vs. +), cell differentiation (WD + MD vs. PD), perineural invasion (No vs. Yes), and stage (I/II vs. III/IV) were not associated with TERT promotor mutation (Supplementary Table S2).

### TERT promoter mutations and prognosis

Considering the heterogeneity in data to evaluate prognostic value of TERT promotor mutation, we decided to conduct the summary synthesis without meta-analysis for prognosis outcome. Only two studies, Chang et al.^16^ and Yilmaz et al.^24^ provided survival analysis for TERT promotor mutation in OSCC. Other studies either provide OS for all tumours inclusive of OSCC or OSCC with either oropharynx, hypopharynx, tonsil and/or larynx. These did not perform the OSCC specific survival analysis for TERT mutation promotor. According to Chang et al.^16^, overall survival, disease free disease-specific survival (DSS), and disease-free survival (DFS) (5 year) for C228T and C250T was 71.1%, 74%, 663% and76.9%, 849%, 807%, repectively. Yilmaz et al.^24^ reported 110 − 109 (11.84-118.16 and 96.62-121.38) month median overall cumulative survival rate.

### Risk of bias and quality assessment

**Fig. 5:**
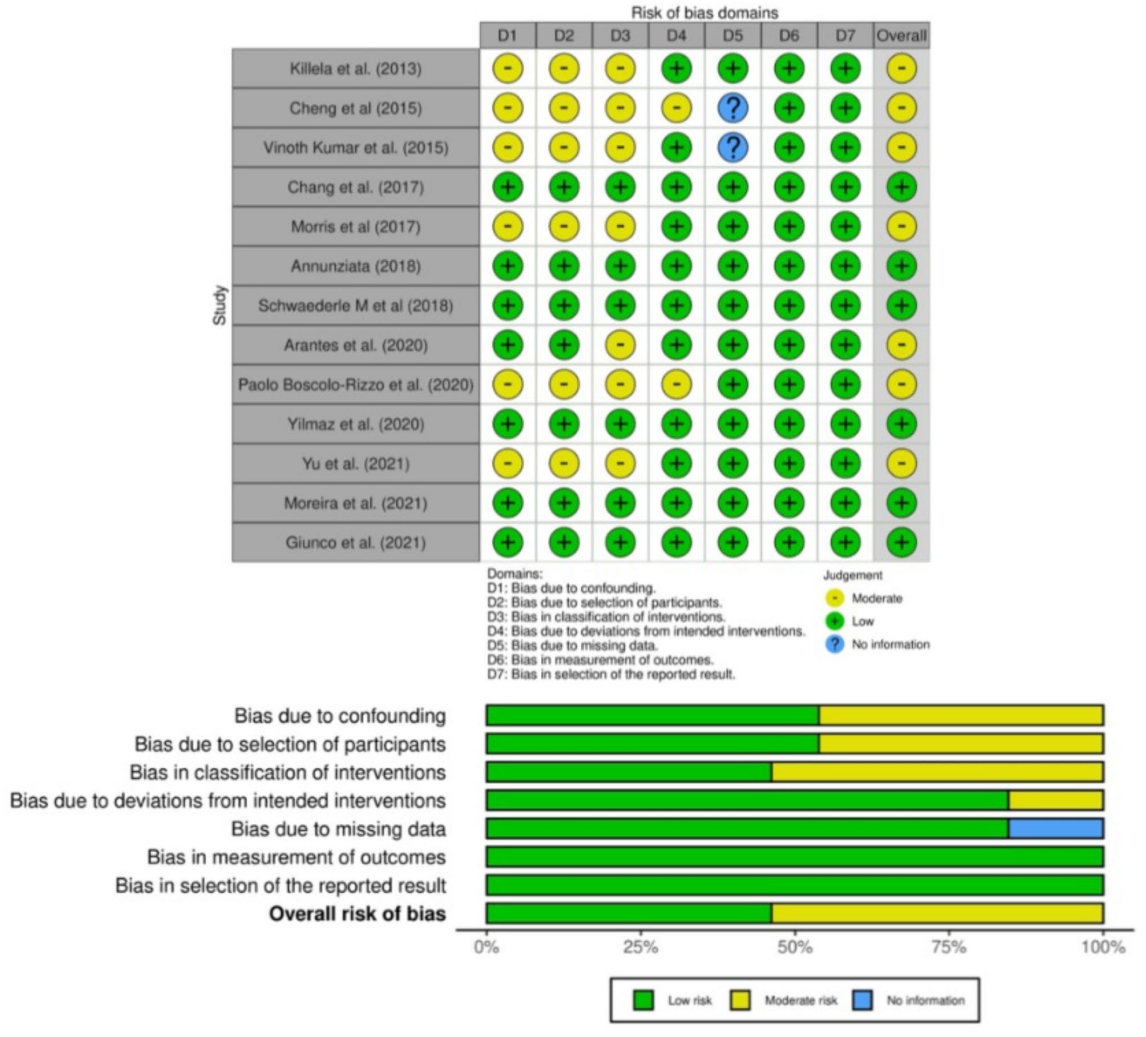
Risk of bias graph for included studies.

The risk of bias assessment is presented in Fig. 5. Six studies were low risk of bias whereas 7 studies were graded as moderate risk mainly due to bias in controlling the confounding factors that could have impacted their analysis.

## Discussion

The TERT enzyme is an essential component in normal cell growth and development. Several tumours have reported the mutation in TERT promotor, altering cell division and survival. The present systematic review found an association between OSCC and TERT promoter mutations, which is consistent with a previous published work on telomerase enzymatic activity in OSCC^23^. There is three-fold high risk of OSCC development in the presence of TERT promotor mutation. TERT mutation OSCC has a pooled frequency of 41.44% (0.41, 95% CI, 0.28, 0.56), which is slightly lower than the majority reported in the literature. However, this difference is due to variation in the site of involvement in oral cavity as well as geographic location. Several other studies reported TERT promotor mutations in OSCC in the range of 17 to 81.1% In the present systematic review, pooled prevalence range was 31%to 60% Giunco et al.^30^. reported a 31.3% (45/144 cases) TERT promotor mutation in their study. Furthermore, Yilmaz et al.^24^ and Yu et al.^23^. found TERT promotor mutation in 75% and 70% of their study cohorts, respectively, which is higher than the reported range in the literature^30^. Clinical and pathological difference among the cohort could be a major reason for the higher frequency of TERT promotor mutation in these two studies, which needs further evaluation.

TERT promotor mutations have been negatively correlated with tumor clinicopathological features in other tumors such as melanoma, bladder carcinoma, gliomos, and thyroid carcinoma^31–34^. In a meta-analysis of TERT promoter mutations in papillary thyroid carcinoma, it was found that they are closely associated with aggressive clinicopathological factors and poorer prognosis. Tumors with TERT promotor mutations are more likely to have a larger tumor, recurrence, vascular invasion, and local node metastasis^31^. TERT promotor mutations were commonly associated with OSCC as compared to other head and neck tumors. The high turnover rate of the oral mucosa may be a factor in the incorporation of mutation in these cells. Unfortunately, tumour tissue derived from tissue with a high turnover rate has a lower TERT promotor mutation frequency, whereas tumour tissue derived from tissue with low self-renewal, such as the tongue, has a higher TERT promotor mutation frequency^31^. Oral mucosa have different turnover rate such as buccal mucosa and gingival have high turnover rate whereas tongue and palate have low turnover rate. This differential expression pattern needs further evaluation. Further, OSCC with TERT promotor mutation also have an increased risk of local regional spread, which can be correlated with increased tumor size due to increased proliferation of tumor cells. However, Paolo Boscolo-Rizzo et al.^15^ suggested that instead of TERT promotor mutation, it is the TERT expression that can affect the clinical outcome. Further, they reported no association of TERT promotor mutations and survival status, which is contradictory to what has been reported in the literature. Giunco et al.^30^ reported higher risk of local recurrence, death, and disease progression. This indicate that different mechanism may be involved tumor proliferation. Between C250T and C228T, C228T hotspot mutation was associated with decrease disease-free survival and overall survival^28^. In addition, the C228T mutation was significantly associated with betel nut chewing^16,28^ whereas C250T mutation with alcohol consumption^24^. Because most OSCC patients chew tobacco and betel nuts, especially in Southeast Asia, the association of habits with specific hotspot mutations could be intriguing.

TERT promoter mutations are commonly reported at specific hotspots within the TERT promoter region, particularly at positions C228T and C250T. These two hotspots have been frequently associated with increase telomerase activity and tumors including OSCC which is consistent with the present study findings^34^. We observed the presence of C228T hotspot mutations in the majority OSCC as compared to C250T, suggesting a differential mechanism in regulation and expression of these hotspot mutations. Similar results were observed by Annunziata et al.^26^, Morris et al.^21^, Vinothkumar et al.^25^, Chang et al.^16^ study. In contrast, Barczak et al.^35^ and Arantes et al.^28^ found C250T more frequently in OSCC. There is no clear explanation for mutation at these specific two hotspots in the chromosome. Epigenetic factors have been suggested to play a role in these mutations through histone modification or DNA methylation. Further, GABP or other ETS family transcription factors have been closing linked to TERT promotor mutation^36^. Concurrence of rs2853669 Polymorphism, BRAF V600E mutation, and TP53 mutation has been reported^37^.

There were some limitations in this meta-analysis. First, most studies were designed retrospectively, which may cause potential selection bias for better-documented patients and larger tumors, since they were more available for collection and analysis. Second, heterogeneity was present in some analyses, probably due to confounding factors, such as patient demographics, ethnicity, sample source, therapeutic approaches, duration of follow-up, and so forth. Further studies have found an association with prognosis and recurrence. Lastly, the sample sizes of some included articles are relatively small, and relevant unpublished data could not be obtained for further analysis. Therefore, our conclusions should be interpreted cautiously considering the heterogeneity and non-availability of comprehensive clinicopathological features data on OSCC in primary studies.

## Conclusion

This meta-analysis demonstrated that the association of TERT promoter mutations with OSCC. Further, this meta-analysis demonstrated that TERT promoter mutations could be considered as biomarkers assisting in risk stratification and prognostic prediction. While these mutations were not associated with certain clinical parameters such as gender, smoking, betel nut use, nodal involvement, metastases, or TNM stage, they remain noteworthy as potential biomarkers. Clinicians can use information about TERT promoter mutations to counsel patients about their prognosis and potential disease course. This can facilitate informed decision-making and psychological support. This systematic review underscores the importance of further research to elucidate the specific role and clinical implications of TERT promoter mutations in the context of OSCC. Future studies should focus on the effect estimated the occurrence of OSCC in individuals with exposure (TERT mutation) versus individuals without exposure (absence of TERT mutation).

## Electronic supplementary material

Below is the link to the electronic supplementary material.

Supplementary Material 1

## Data Availability

All data generated or analysed during this study are included in this published article and its supplementary information files.
