# Supplementary material for: Association of TERT promoter mutation with oral squamous cell carcinoma: a systematic review and meta-analysis
Source: Sci Rep. 2025 Apr 30;15:15257. doi: 10.1038/s41598-025-98133-6 (PMC12043803; doi:10.1038/s41598-025-98133-6)
Supplement: Supplementary file 1 — Supplementary Material 1 [file 41598_2025_98133_MOESM1_ESM.pdf]

Monal Yuwanati, Sachin Sarode, Gargi Sarode, Amol Gadbail, Shailesh Gondivkar, Akhilanand Chaurasia, Dhara Dwivedi, Sara Delgadillo-Barrera. Association Of TERT Promoter Mutation with Oral Squamous Cell Carcinoma: A Systematic Review and Meta-Analysis

Supplementary Information

Supplementary Table S1: PECO Framework

Condition or domain being studied	Squamous cell carcinoma of oral cavity
Participants/population	Patients with histologically confirmed oral squamous cell carcinoma
Exposure(s)	TERT promoter mutations
Comparator(s)/control	Oral squamous cell carcinoma with and without TERT mutations, or normal oral mucosa
Main outcome(s)	The proportion and/or prevalence of TERT promoter mutation in OSCC patients.
Secondary Outcome	Any other
Study Design	Case-control, cohort, cross-sectional studies

Supplementary Table S2- Supporting data for supporting the statement on clinicopathological correlation with TERT Mutation

Clinicopathological Parameters		Total	TERT +	TERT - / Wild	Odds Ratio (OR)	96% CI	p value
**Site**							
	Tongue	172	83	89			0.55
	Alveolar arch	5	3	2			0.80
	Floor of Mouth	53	19	34			0.35
	Buccal	31	19	12			0.22
	Retromolar trigone	4	2	2			0.84
	Gingiva	26	7	19			0.18
	Other	14	5	9			0.66
**Gender**							
	Female	126	68	58	1.21	(0.80 to 1.82)	0.425
	Male	321	158	163			
**Smoking Habit**							
	Smokers	169	77	92	0.76	(0.51, 1.11)	0.163
	Nonsmokers	271	142	129			
**Alcohol**							
	Drinker	230	107	123	0.76	(0.52, 1.10)	0.154
	Non-drinkers	210	112	98			
**Age**							
	<50	88	45	43	0.80	(0.49, 1.29)	0.431
	>50	254	144	110			
**Betel Nut Chewer**							
	Chewer	156	89	67	2.66	(1.32 to 5.32)	**0.005***
	Nonchewer	45	15	30			
**T stage**							
	T1+T2	181	75	106	0.86	(0.57, 1.32)	0.490

	T3+T4	164	74	90			
**Node status**							
	N -	226	99	127	1.08	(0.69, 1.68)	0.750
	N +	119	50	69			
**Extracapsular spread**							
	(-)	144	122	166	0.82	(0.46, 1.44)	0.059
	(+)	57	27	30			
**Cell differentiation**							
	WD + MD	175	91	84	1.08	(0.47, 2.47)	0.849
	PD	26	13	13			
**Perineural invasion**							
	No	114	63	81	0.87	(0.51, 1.48)	0.617
	Yes	87	41	46			
**Stage**							
	I-II	75	26	49	1.40	(0.68, 2.8)	0.356
	III-IV	69	19	50			

*Significant
